# One year of free school fruit in Norway – 7 years of follow-up

**DOI:** 10.1186/s12966-015-0301-6

**Published:** 2015-11-10

**Authors:** Elling Bere, Saskia J. te Velde, Milada Cvancarova Småstuen, Jos Twisk, Knut-Inge Klepp

**Affiliations:** Department of Public Health, Sport and Nutrition, University of Agder, Postboks 422, 4604 Kristiansand, Norway; Department of Epidemiology and Biostatistics, VU University Medical Centre, Amsterdam, The Netherlands; Department of Nutrition, University of Oslo, Faculty of Medicine, Oslo, Norway

**Keywords:** Fruit and vegetable, School-based intervention, Long-term follow-up

## Abstract

**Background:**

It is important that health-promoting efforts result in sustained behavioural changes, preferably throughout life. However, only a very few intervention studies evaluate long term follow up.

**Objective:**

The aim of the present study is to evaluate the overall and up to seven years effect of providing daily one piece of fruit or vegetable (FV) for free for one school year.

**Methods:**

A total of 38 randomly drawn elementary schools from two counties in Norway participated in the Fruit and Vegetables Make the Marks project. Baseline (2001) and follow-up surveys were conducted in May 2002, 2005 and 2009 (*n* = 320 with complete data) to assess FV and unhealthy snack intake. Mixed models were used to analyze the data.

**Results:**

Statistically significant adjusted overall effects of the intervention were revealed for FV intake (1.52 times/day) but this weakened over time. A significant adjusted overall effect (−1.54 consumptions/week) and a significant seven-year-follow-up effect (−2.02 consumptions/week) was found for consumption of unhealthy snacks for pupils of parents without higher education.

**Conclusion:**

One year of free school fruit resulted in higher FV intake and lower unhealthy snack intake, however this weakened over time for FV intake and became stronger for snack intake. More follow-up studies with larger samples and lower attrition rates are needed in order to further evaluate the long-term effect.

## Background

A diet high in fruits and vegetables (FV) is inversely related to several chronic diseases [1], and an increased intake would improve global public health [2]. In Norway, children and adolescents consume only about half of the national five-a-day recommendation [3]. As food preferences and habits established in childhood to a large extent tend to be maintained into adulthood [4, 5], and in order to achieve maximum prevention potential, it is important to get children to eat more FV.

It is also important that effective efforts conducted to increase children’s FV intake result in sustained elevated FV intakes, preferably throughout life, in order to have a maximum health prevention potential. However, only a few school-based intervention studies evaluate follow-ups longer than one year after the end of the intervention [6] or used epidemiological modeling to estimate long-term effects [7]. The evaluation of the school fruit schemes in Norway, within the Fruits and Vegetables Make the Marks (FVMM) project, is one of very few studies that had a significant impact on FV consumption, and also an elevated intake for longer time periods than one year after the intervention: One free piece of fruit or vegetable every school day for one school year increased FV intake by 0.9 portions/day while the program was running [8], and the elevated effects one and three years after the end of the intervention were respectively 0.5 and 0.4 portions/day, compared to controls [9, 10]. However, parts of the sustained effect may have been due to the fact that intervention pupils had an increased participation in the Norwegian school fruit subscription program [9, 10], a program that since 2003 has been available for all Norwegian elementary and secondary schools. It is therefore important to assess the sustained effect also when the participants have finished school and are not provided with fruit anymore.

The free fruit program also resulted in a decreased consumption of unhealthy snacks (scale of soft drinks, candy and potato chips) measured both while the program was running [8] and one year after the end of the intervention [11]. This effect had, however, lost its statistical significance three years after the end of the intervention [10]. Two separate studies have shown that free school fruit reduces the consumption of unhealthy snacks more among pupils of lower socio economic status (SES) than pupils of higher SES [8, 12], i.e. reducing social inequalities as Norwegian children from lower SES families tend to consume less fruits and vegetables [13].

The aim of the present study is to evaluate the overall effect (i.e. average effect) and effects at the specific follow-up moments (i.e. at the end of the intervention (2002), and respectively three years (2005) and seven years (2009) after the intervention) of a project providing one piece of fruit or vegetable for free every school day for one school year on (1) FV intake and (2) consumption of unhealthy snacks.

## Methods

### Design and study sample

A total of 38 randomly drawn elementary schools from two different counties (Hedmark and Telemark) participated in the FVMM project. Nine schools within one of the counties (Hedmark) were randomly selected as intervention schools and participated in the Norwegian School Fruit Program for free (Free fruit group) during the school year 2001/2002. The subscription program was initiated by the health authorities in 1996 and made nationwide in 2003 in collaboration with the Norwegian Marketing Board for Fruits and Vegetables. In this program the schools initially choose to participate or not, and then the pupils at the participating schools can decide to subscribe or not. The cost for the parents is currently NOK 2.50 per school day (approximately EUR 0.30). The pupils who subscribe receive a piece of fruit or a carrot each school day, usually in connection with their lunch meal. The free program was just the subscription program offered for free (no parental payment) for one school year for the 9 schools in the FVMM project. All pupils at these schools received a piece of fruit or a carrot each school day, usually at lunch time. Apples, pears, bananas, oranges, clementines, kiwis, carrots and nectarines were the most frequently FV given. The free program started in October 2001, and lasted throughout the school year (i.e. until June 2002). In the present study, the remaining 29 schools served as control schools. The control group thereby also included schools that from the school year 2001/2002 participated in the Norwegian school fruit subscription program (paid version) (9 schools in which 41 % of the pupils subscribed in the school year 2001/2002) [8]. Some of the pupils (6th graders at nine schools in both counties, but no 7th graders) did also receive a FV educational program during the intervention year (2001/2002). In the study sample used in the current paper, 63 pupils of the intervention group (56 %) and 38 pupils of the control group (18 %) received the education program. This educational program showed, however, no effect on increasing FV intake [9, 14], and all pupils in the FVMM cohort are therefore included in the present study to increase the statistical power.

A baseline questionnaire survey was conducted in September 2001, while follow-up surveys were conducted in May 2002, May 2003 May 2005 and September 2009. The effect of the free fruit intervention has been reported for the 2002 [8], 2003 [9] and 2005 [11] surveys. The present study reports the average seven year follow-up effect including data from the 2001 baseline survey, and the 2002, 2005 and 2009 follow-up surveys. Data from the 2003 survey is not included because it only included half of the study population (i.e. only the initial 6^th^ graders), due to the fact that the initial 7^th^ graders were mostly at different schools (in Norway 8^th^ grade is a secondary elementary school, different from the primary elementary school). In September 2001, all 6th and 7th graders at the included schools were invited to participate. In September 2009 most of them had finished high school, i.e. all with normal school progression.

### Ethics, consent and permissions

Informed consent was sought from both the children and their parents prior to the study. Ethical approval and research clearance for this project was obtained from The National Committees for Research Ethics in Norway and from The Norwegian Social Science Data Services [file number 12395].

The FVMM cohort includes 1950 pupils (participants at baseline): 984 boys and 966 girls, 585 in the Free fruit group and 1365 in the Control group. Average age was estimated to be 11.8 years at baseline. A total of 320 pupils (16 %) also participated at the follow-up survey in September 2009, and constitute the study sample for the present study. Of these respectively 296 and 282 participated in the 2002 and 2005 surveys. Descriptive data of the study sample are presented in Table 1. At baseline 296 of these pupils had a parent/guardian who completed a parent questionnaire. The average parental age was 40.5 years, and 86 % of the parents were mothers/female guardians.

**Table 1:** Baseline characteristics of the current study sample and those lost to follow-up

	Attrition analyses			Current study sample		
	Lost to follow-up	Current study sample	*p*-value*	Free fruit group	Control group	*p*-value*
Number	1630	320		112	208	
Sex (% girls)	47	62	<0.001	59	64	0.355
Class grade (% 7th grade)	47	48	0.651	44	51	0.218
Parental education (% high)	40	49	0.009	53	46	0.229
Group (% free fruit pupils)	29	35	0.033			
	Mean ± SD	Mean ± SD	*p*-value**	Mean ± SD	Mean ± SD	*p*-value**
FV intake baseline (portions/day)	2.4 ± 2.4	2.5 ± 2.6	0.900	2.2 ± 2.2	2.6 ± 2.7	0.123
FV intake baseline (times/week)	14.0 ± 7.2	14.5 ± 6.9	0.271	13.7 ± 7.4	15.0 ± 6.5	0.127
Unhealthy snacks baseline (times/week)	7.2 ± 4.6	6.5 ± 4.1	0.009	6.7 ± 3.9	6.3 ± 4.2	0.500

*based on Chi square test

**based on independent t-test for continuous data

SD = Standard Deviation

### Instruments

A survey questionnaire was completed in 2001, 2002 and 2005 by the pupils in the classroom in the presence of a trained project worker. The 2009 survey was sent by regular mail. A written 24-h FV recall was used to assess pupils’ FV intake (portions/day) [15]. FV intake the previous day was recorded for school days for the 2001, 2002 and 2005 surveys (i.e. the survey was conducted on weekdays, Tuesday through Friday). In 2009 we did not specify for which days the questionnaire should be completed, and therefore the data includes both weekdays and weekend days. In the 2001, 2002 and 2005 surveys, the 24-h recall separated the day into five time periods (before school, at school, after school, at dinner and after dinner). In 2009, as most participants had finished school, the day was separated into four time periods, which were more applicable to adults: breakfast, lunch (between breakfast and dinner), dinner, and supper (after dinner). FV intake for the full day was calculated (portions/day). In addition, usual FV intake was measured with four food frequency questions (FFQ, times/week), and unhealthy snacks consumption with three food frequency questions (soda/candy/potato chips, times/week). Both the 24-h recall and the food frequency questions have been presented previously, and their validity and reliability have been reported for FV intake among 6th graders. The instrument was found to have good test-retest reliability [12, 15] . In a validation study, the 24-h recall part of the questionnaire gave valid estimates for the average intake of vegetable, but overestimated the intake of fruit and juice, while the ability to rank subjects according to intake of fruit and vegetable based on the frequency part was rather low, however similar to other studies, compared to a reference method [15]. The pupils reported their own sex and parents recorded their level of education at baseline (lower: no college or university education vs. higher: having attended college or university).

### Statistical analyses

Descriptive statistics were used to characterize the sample. Comparisons were made between those participating in the baseline and the 2009 survey and those participating in the baseline survey but not in the 2009 survey (lost to follow-up, attrition). Additionally, comparisons were made between the intervention and the control group. For these comparisons independent T-tests for continuous variables and chi-square statistics for the categorical variables were used (Table 1).

For all three outcomes (FV all day (portions/day), usual FV intake (times/week) and soda/candy/chips (times/week)) linear mixed models (including all three post intervention measures as outcome) were used to evaluate the overall (i.e. average) effect of the intervention and its effect on the three different follow-up assessments separately. Both a crude analysis (only adjusted for the baseline value of the particular outcome and including random intercepts to account for the nested design (assessments within participants, participants within school), model 1) and an adjusted analysis (further adjusted for sex and parental education, model 2) was performed. The overall intervention effect was estimated in a model without the time variable, as it was not a confounder in the association [16], in order to estimate the average effect over the whole follow-up period. To estimate intervention effects at specific follow-up assessments, time and interaction terms between time and group (intervention vs control) were included in the models. Time was included in the models as a categorical variable as no linear effect over time was expected. Regarding the model for unhealthy snacks, the results were a priori stratified by parental education. This is based on the earlier finding that parental education was a significant effect modifier in this association, and that only among pupils of parents without higher education a significant effect on snack intake was found [8, 12]. Contrary, a previous study in the same cohort found that parental education was not an effect modifier for the intervention effects on FV intake [8].

Due to the design of the study, actually four intervention conditions could be distinguished, i.e. 1) no intervention; 2) education program only; 3) free FV only; 4) education program + free FV. Therefore, sensitivity analyses were conducted by distinguishing four intervention conditions. However, due to lack of power, only overall effects were estimated.

An examination of the residuals did not reveal unacceptable departures from normality. All analyses were conducted using IBM SPSS statistics 22 (IBM Corporation, Armonk, NY, USA), and all tests were two-sided with p-values < 0.05 considered statistically significant.

## Results

In total, we analyzed 320 pupils with an estimated mean age of 11.8 years at baseline; 62 % girls, 49 % with parents with higher education, 48 % 7^th^ graders, and 35 % of the study sample were in the free fruit intervention group (Table 1a). Compared to those lost to follow-up, the current study sample included more girls, pupils of parents with higher education, participants from the intervention group, and reported a significantly lower consumption of unhealthy snacks at baseline (Table 1). There were no statistically significant differences in the reported baseline characteristics between the intervention and the control group in the current study sample (Table 1).

Observed mean values for fruit and vegetable intake and consumption of unhealthy snacks for all survey points are presented in Table 2, while estimated overall and time specific intervention effects are presented in Table 3.

**Table 2:** Observed means, standard deviations (SD), medians and interquartile range (IQR) for fruit and vegetable (FV) intake (portions/day and times/week) and consumption of unhealthy snacks (times/week) at all surveys

Year	2001				2002				2005				2009			
	mean	SD	median	IQR	Mean	SD	Median	IQR	mean	SD	median	IQR	mean	SD	Median	IQR
FV all day (portions/day)	2.2	2.2	1.5	0.0 - 3.0	2.5	2.3	2.0	1.0 -3.8	2.7	2.2	2.3	1.0 - 4.0	2.3	2.0	2.0	1.0 - 3.5
Intervention	2.6	2.7	2.0	1.0 - 3.5	1.9	1.8	1.5	0.50 - 3.0	2.5	2.9	2.0	0.0 - 4.0	2.1	1.9	2.0	1.0 - 3.0
Control																
Usual FV intake (times/week)																
Intervention	14	7.4	14	8.0 - 18	15	6.9	16	10 - 19	15	7.2	15	11 - 21	13	7.4	13	7.0 - 17
Control	15	6.5	15	10 - 19	14	6.6	13	8.5 - 19	14	7.6	14	9.0 - 19	13	7.1	11	7.5 - 17
Consumption of unhealthy snacks (times/week)																
Lower parental education																
Intervention	7.5	4.3	6.0	3.0 - 9.3	6.1	3.5	5.3	3.1- 8.0	6.2	5.7	5.0	3.0 - 8.0	5.0	3.5	4.0	2.5 - 6.6
Control	6.0	3.2	5.0	4.0 - 8.3	6.2	3.7	6.0	6.0 - 7.5	6.4	4.7	5.0	3.0 - 8.8	6.1	4.7	5.0	2.7 - 8.0
Higher parental education																
Intervention	5.6	2.9	5.0	4.0 - 5.0	5.5	3.2	5.0	3.5 - 7.0	5.0	3.3	4.0	2.6 - 6.5	4.5	3.5	4.3	2.1 - 6.0
Control	6.7	5.1	5.0	3.0 - 5.0	6.7	5.1	5.0	3.0 - 84	5.9	4.2	4.5	3.0 - 7.4	4.6	3.5	4.0	2.0 - 6.0

**Table 3:** Estimated overall intervention effects (regression coefficients (b) with 95 % confidence intervals (CI)) for fruit and vegetable intake and consumption of unhealthy snacks and for each follow-up assessment based on mixed models

	model 1^a^				model 2^b^			
	b	95 % CI		*p*-value	b	95 % CI		*p*-value
Fruit and vegetable intake (portions/day)								
Overall effect	0.44	(0.10;	0.77)	0.012	0.34	(−0.01;	0.68)	0.057
Follow-up 1 (2002)	0.65	(0.14;	1.16)	0.012	0.51	(−0.02;	1.04)	0.059
Follow-up 2 (2005)	0.28	(−0.24;	0.79)	0.289	0.15	(−0.38;	0.68)	0.572
Follow-up 3 (2009)	0.35	(−0.13;	0.84)	0.152	0.31	(−0.19;	0.82)	0.223
Fruit and vegetable intake (times/week)								
Overall effect	1.31	(0.20;	2.43)	0.021	1.52	(0.40;	2.65)	0.008
Follow-up 1 (2002)	1.38	(−0.21;	2.97)	0.089	1.53	(−0.06;	3.11)	0.059
Follow-up 2 (2005)	1.74	(0.14;	3.33)	0.033	1.99	(0.40;	3.58)	0.014
Follow-up 3 (2009)	0.88	(−0.63;	2.39)	0.254	1.09	(−0.42;	2.60)	0.158
Unhealthy snacks (times/week) Lower educated								
Overall effect	−1.23	(−2.24;	−0.22)	0.017	−1.54	(−2.54;	−0.53)	0.003
Follow-up 1 (2002)	−0.78	(−2.28;	0.71)	0.302	−0.87	(−2.32;	0.57)	0.234
Follow-up 2 (2005)	−0.97	(−2.46;	0.52)	0.202	−1.65	(−3.09;	−0.20)	0.026
Follow-up 3 (2009)	−1.88	(−3.31;	−0.45)	0.010	−2.02	(−3.41;	−0.64)	0.004
Higher educated								
Overall effect	−0.21	(−1.06;	0.63)	0.617	−0.22	(−1.06;	0.62)	0.606
Follow-up 1 (2002)	−0.51	(−1.72;	0.70)	0.409	−0.53	(−1.74;	0.69)	0.395
Follow-up 2 (2005)	−0.60	(−1.82;	0.61)	0.331	−0.50	(−1.71;	0.72)	0.425
Follow-up 3 (2009)	0.44	(−0.72;	1.61)	0.445	0.35	(−0.82;	1.52)	0.558

^a^adjusted for baseline intake and with random intercept for individual and school

^b^further adjusted for sex and parental education

### FV intake

As can be seen in Table 3 in the crude analysis a statistically significant overall effect of the intervention of 0.44 portions of FV per day and 1.31 times a week was found (model 1). The effect remained significant after adjustment for sex and parental education (model 2) for FV intake assessed by FFQ (1.52 times/week) but not for FV intake assessed by 24-h recall (borderline significant, *p* = 0.057). The effect of the intervention on FV intake assessed by 24H-recall weakened over time, from 0.65 portions/day (*p* = 0.012, model 1) at 1^st^ follow-up to 0.28 portions/day (*p* = 0.289, model 1) and 0.35 portions/day (*p* = 0.152, model 1) at 2^nd^ and 3^rd^ follow-up, respectively. For FV intake assessed by FFQ, the effect was strongest at 2^nd^ follow-up, 1.74 times/week (*p* = 0.033, model 1), but weakest at 3^rd^ follow-up (0.88 times/week, *p* = 0.254, model 1). Effects for FV assessed by 24-hour recall weakened after adjustment for sex and parental education (model 2), while they strengthened for FV assessed by FFQ.

### Unhealthy snack intake

A significant overall reduction of 1.23 times/week was found for the consumption of unhealthy snacks for pupils of lower educated parents, but not for pupils of higher educated parents. This effect remained significant after adjustment for sex (−1.54 times/week, *p* = 0.003, model 2). The effect became stronger over time as indicated by statistically significant effects at the 2^nd^ follow-up of −1.65 times/week (*p* = 0.026, model 2 only) and 3^rd^ follow-up of −2.02 times/week (*p* = 0.004, model 2, Table 3).

### Sensitivity analyses

Results from the sensitivity analyses distinguishing four intervention conditions showed that in general for fruit and vegetable intake, the effect size of the group receiving both the free fruit and the education program was largest and that the effect size of those who received the education program only was not statistically significant, i.e. their intake did not differ from the reference group (receiving no free fruit and no education program). Only for snack intake among children from higher educated parents, the pattern was different; effect size was biggest for those who only received the education program, but it did not differ from the reference group (results not shown).

## Discussion

In the present study, significant overall effects of the free fruit intervention were observed for FV intake (1.52 times/week) and for unhealthy snacks among pupils of low SES (−1.54 times/week). The effect on FV intake weakened over time and was not significant anymore at seven years after the intervention. Interestingly, the effect on unhealthy snacks became stronger over time with a significant favorable effect at the last follow-up.

School fruit schemes, similar to the present free fruit program, also exist in several other countries. Review studies show that such schemes in general are effective in increasing fruit intake by 0.14 – 0.99 portions a day while in operation [17, 18]. Except from the present Norwegian long term effect evaluation, only a few studies evaluate the effect of the schemes after the children stop receiving free school fruit. Evaluations of the UK school fruit scheme have reported no significant effect when the pupils were no longer eligible to receive free fruit [19–22], or even negative effects [21]. Lasting effects are important for assessing the potential health effect of the schemes. Like in the UK school fruit scheme, the effect on fruit and vegetable intake of the Norwegian school fruit program was not significant seven years after the program ended, however, the estimated effect of a third portion a day can still be relevant for public health. It has been reported that the Norwegian School Fruit Program, offered for free for all 10 compulsory school years, would be cost effective if it resulted in a sustained (lifelong) increase of only 2.5 g per day (conservative estimate) [23], which is less than the observed long term effect in the current study.

The results of the present study indicates an average reduction of 1.54 consumptions of unhealthy snacks a week after exposure to one year of free school fruit in the low parental education group but not in the high parental education group. This non-intended effect of the free fruit program has been reported earlier both while the program was running [8], and one year after the end of the program [11]. Interestingly this effect increased over time, which could be chance finding or be explained by the fact that the newly achieved habit is sustained. Analyses of the Norwegian data from the Health Behavior in School-aged Children (HBSC) in the same time period did not find differences in time trends by socio-economic status, but did find an overall healthier diet over time with more fruits and vegetables and less sweets and sugar drinks [13]. Recently, a decrease in consumption of unhealthy snacks in low SES children also has been reported due to the national free school fruit program that has been implemented in Norway in all secondary and combines elementary and secondary schools from 2007 to 2014 [12]. Also the Dutch Schoolgruiten project, providing school children with one piece of fruit or ready-to-eat vegetables for free twice a week, reported that a school fruit scheme reduced unhealthy snacking (during school breaks) [24]. Replacing snacks by fruits and vegetables can be explained by the behavioral choice theory; people make choices among alternatives and when fruits and vegetables are easily assessable and for free, they may become a good alternative. Moreover, when fruits and vegetables are easily available, the need for energy is satisfied and there is no need for energy from snacks. However, this theory can explain the short term effects, immediately after the intervention, but not necessarily the long-term effects. Habit theory [25, 26] may be another explanation; repeatedly performing a specific behavior, i.e. consuming fruits or vegetables during breaks, may induce habit formation by which the behavior becomes automatic or habitual [25, 26]. Once the consumption of fruits and vegetables during breaks has become a habit, this behavior will be sustained. That this effect was only observed in participants from lower educated parents might be explained by the fact that they had a higher intake at baseline (Table 2) and therefore had more room for improvement.

The major limitations with the present study is the low participation rate in the 2009 survey (defining the present study sample) as only 16 % of the original cohort participated, and the study sample was clearly a selective sample. This lowering of statistical power and the biased sample might have influenced the result, making it difficult to conclude about the long term effect. We can only speculate about the direction of the bias, the drop-outs did not differ from the ones that stayed in the study with respect to baseline intake of fruit and vegetable, but females and those from higher educated families were more likely to stay in the study. From previous research (e.g. [13]) we know that females and higher educated people have in general more healthy diets. In addition, participants from the intervention group were more likely to stay in the study, therefore, observed effects may be overestimated. It may also explain the discrepancies with earlier analyses in the same cohort. In addition, a second limitation is the design of the study: Some pupils in the control group (38, 18 %) and in intervention group (63, 56 %) also received a FV educational program during the intervention year (2001/2002) [9, 14]. Results of the sensitivity analyses indicate that the fact that some pupils in both the intervention and the control groups received an education program may have influenced the findings; there might be a synergic effect of providing both free fruit and an educational program. Another limitation was the fact that the 2009 questionnaire differed slightly from the previous questionnaires and 24 h recall reports may have included weekend days during which people tend to eat less healthy [27, 28]. Therefore, FV intake levels in 2009 may have been underestimated, however this is likely the same for the intervention and control group.

## Conclusion

The results of the present study indicate a significant and relevant overall effect of free school fruit on FV intake and for unhealthy consumptions in children from parents without higher education. The effect weakened for FV intake, but increased for unhealthy snack consumption, with time. Because of the current selected sample and a very low participation rate at the last survey, more follow-up studies are needed in order to further evaluate the long-term effect of FV interventions.

## Abbreviations

24 h-recall: 24 h recall
95%CI: 95 % Confidence Interval
b: regression coefficient
FFQ: food frequency questionnaire
FV: fruit and vegetable
FVMM: fruit and vegetables make the marks
SES: socio-economic status
